# Dr. Matthew D. Mann

**Published:** 1910-07

**Authors:** 


**A Monthly Review of Medicine and Surgery.**

*EDITOR*

WILLIAM WARREN POTTER, M. D.

All communications, whether of a literary or business nature, books for review and xchanges, should be addressed to the editor, 238 DELAWARE AVENUE, BUFFALO, N.Y.

VOL. LXV. JULY, 1910. No. 12

### Dr. Matthew D. Mann

ONE of the most interesting events in the medical history of Buffalo took place on the evening of May 26, 1910, it being in the nature of a complimentary dinner to Professor Matthew D. Mann, tendered by the Alumni Association of the Medical Department of the University of Buffalo. When it became known a short time ago that Dr. Mann would retire from the chair of obstetrics and gynecology at the close of the academic year ending May 27, 1910, the alumni association took the matter up through its executive committee, and after some discussion it was determined that the testimonial should take the form of a dinner, to be served at the Lafayette Hotel on the date already mentioned.

The invitations were liberally responded to, and on the evening in question about two hundred alumni and alumnæ assembled to do honor to the famous teacher, the women numbering about twenty.

Seats were taken at 7.30, when the service began. The menu was:

| | |
|---|---|
| Little Neck Clams on Halfshell | Bronx Cocktail |
| Olives Celery | |
| Clear Mock Turtle au Madeira | |
| Filet of Sole au Vin Blanc | |
| Potatoes Hollandaise | |
| Fresh Mushrooms Sur Cloche | |
| Broiled Spring Chicken au Cresson | Rhinewine Punch |
| Potatoes Rissolle | |
| Tomatoes Surprise | |
| Frozen Fruit Biscuit | |
| Assorted Cakes | |
| Coffee | Cigars Cigarettes |

These were the regular toasts:

Toastmaster .................................... Dr. Charles G. Stockton

"He was a Mann, take him for all in all,
I shall not look upon his like again."

Mann as Writer.................................Dr. James W. Putnam
"Arma virumque cano"
Mann as Teacher, a tribute from his Students..........................
...........................Clayton M. Greene, for Class of 1910
"And still they gazed and still the wonder grew
That one small head could carry all he knew."
Mann as Surgeon...................................Dr. W. E. Ford
"A wise physician, skilled our wounds to heal,
Is more than armies to the public weal."
Mann as Citizen.................................Dr. Thomas H. McKee
"Principle is ever my motto, not expediency"
Mann—Reminiscence..............................Dr. James S. Smith
"In all thy humors, whether grave or mellow,
Thou'rt such a touchy, testy, pleasant fellow"
Mann—a word in recognition............................Dr. F. C. Busch
"There is nothing so strong or safe in any emergency
of life as the simple truth."
In Reply..........................................Dr. M. D. Mann
"I count myself in nothing else so happy
As in a soul remembering my good friends."

Dr. Stockton, always at home as the master of ceremonies, was at his best on this occasion. Paraphrasing the sentiment attached to his office, he made it read "He *is* a Mann" and made a felicitous introductory speech full of befitting allusions to the guest of the evening. He then introduced Dr. Park, who entered the room a few moments before.

Dr. Roswell Park, though not on the regular toast list, being convalescent from a serious illness and about to depart for Europe seeking complete restoration to health, nevertheless, insisted in spite of the advice of his physician on being present to pay tribute to his friend and colleague. He referred to Dr. Mann's conscientiousness; the high moral tone which he had established at the university, and the manner in which he weighed the responsibility attached to issuing diplomas to hundreds of students during the last twenty-eight years.

Dr. James Wright Putnam, said it might be inferred from the quotation given with his topic, that he was expected to sing praises of Dr. Mann as a writer; not so, however, but he took delight in saying that to know his writings is to know the history of gynecology and obstetrics for the last quarter of a century. He writes clearly, said Dr. Putnam, and is one who has enunciated principles and laid down laws. His speech was a finished tribute to Dr. Mann's literary and scholastic accomplishments.

Clayton M. Greene, for the class of 1910, which would graduate next day, acquitted himself with distinction in speaking for his class; and in closing presented Dr. Mann with a set of resolutions, handsomely engrossed, bound in seal morocco, expressive of the esteem and affection of the members of the class of 1910.

Dr. W. E. Ford, of Utica, the next speaker, said he brought greetings from the citizens of Dr. Mann's former home. He paid eloquent tribute to Dr. Mann's parents and especially to his mother, who had endeared herself to the people of Utica, through her philanthropic work and great kind-heartedness. Dr. Ford then dealt with the ability of the guest as a surgeon, during the course of which he frequently received the plaudits of his auditors. The speech was both eloquent and appropriate as to manner and material.

Dr. Thomas H. McKee delivered one of his characteristically humorous speeches in part, while in other part he spoke with seriousness, in presenting the citizenship side of Dr. Mann's character. The speaker enumerated almost every civic matter that had received the guest's attention, from the time in his young life when he organised a baseball nine, to the founding of Saint Margaret's school in his later years, emphasising in particular the prominent part he played while park commissioner, and alluding in terms of praise to the sportsmanlike blood in his veins. Dr. McKee omitted to mention but one thing, so far as we know, in which Dr. Mann had been interested, and that is his part in establishing the Guido Chorus, an organisation that does the founder much credit, and contributes to the enjoyment of thousands of people.

Dr. James S. Smith was to have presented a reminiscence of Dr. Mann. He was, however, detained by important matters and Dr. Carlton C. Frederick was invited to fill the vacancy, which he did with capability, delighting his hearers with a charming impromptu speech. Dr. Frederick told of his pleasant relations with the guest of honor from the time the latter came to Buffalo when the speaker was interne at the General Hospital, until the present time, giving personal touches here and there that were interesting.

Dr. F. C. Busch said a few words in recognition of Dr. Mann's worth as a teacher and as a faculty colleague. He concluded by presenting to Dr. Mann on behalf of the faculty, a beautiful silver vase appropriately engraved and filled with roses; also a massive bouquet of cut flowers artistically secured with blue ribbon for Mrs. Mann, who was present with some friends, having come in when the speaking began. The speaker was cheered, the vase was cheered, and the flowers were cheered in turn, as Dr. Busch took his seat.

Dr. Matthew D. Mann was now called to his feet in reply, which at first appeared no easy task. But, directly, it was observed that he got into the current and paddled his canoe with an easy swing to the oar, making a graceful, handsome acknowledgment of all the kind words and deeds of the evening. Among other things, Dr. Mann told the assemblage while he was retiring as a teacher in the university, he did not propose to give up surgical or literary work; that he should still continue to operate when occasion presented, and to write when he had anything of value to communicate. Cheer upon cheer greeted Dr. Mann as he rose to reply, and rounds of applause followed as he took his seat.

And so ended one of the most beautiful testimonial dinners ever given by the medical profession in Buffalo. Doubtless other banquets have been larger and more elaborate, but none that was more appropriately planned or more delicately executed. Indeed, the key note of the dinner was simplicity and its *ultima thule* was affection, a heart to heart feeling between guest and hosts characterising it from first to last.

